# Review of the literature and individual patients’ data meta-analysis on efficacy and tolerance of nitroxoline in the treatment of uncomplicated urinary tract infections

**DOI:** 10.1186/s12879-014-0628-7

**Published:** 2014-11-27

**Authors:** Kurt G Naber, Hiltrud Niggemann, Gisela Stein, Guenter Stein

**Affiliations:** Department of Urology, Technical University of Munich, Ismaningerstr. 22, Munich, 81675 Germany; Statistical Consulting & Data Analysis, Schlehendornweg 24, Jena, 07751 Germany; Rosen Pharma, Kirkeler Str.41, Blieskastel, 66440 Germany; Department of Internal Medicine, University of Jena, Erlanger Allee 101, Jena, 07747 Germany

**Keywords:** Nitroxoline, Cotrimoxazole, Norfloxacin, Uncomplicated urinary tract infection, Meta-analysis

## Abstract

**Background:**

Nitroxoline, a hydroxychinoline derivate, has been used for many years to treat urinary tract infections (UTI). Many uncontrolled, but only few controlled clinical studies have been published. Four so far unpublished, controlled clinical studies were meta-analysed.

**Methods:**

A narrative literature review was performed. In addition the individual patient data (IPD) of 466 females with uncomplicated UTI of four prospective, single blind, randomized, clinical studies with similar protocols using nitroxoline (250 mg tid) versus cotrimoxazole (960 mg bid) or norfloxacin (400 mg bid) as controls for 5 days (sporadic UTI) or 10 days (recurrent UTI) were meta-analysed. The primary aim was eradication of bacteriuria 7–13 days after end of therapy (test of cure). Clinical efficacy was determined by elimination of symptoms and safety by adverse events and laboratory tests.

**Results:**

Reviewing a total of 26 uncontrolled, 2 controlled and one postmarketing studies including more than 11,000 patients, good efficacy and safety of nitroxoline could be confirmed. In the four unpublished controlled studies a total of 234 patients were treated orally with nitroxoline and 232 with controls. The safety of nitroxoline was very good and comparable to the controls (adverse events 9.4% vs 7.8%; p = 0.360). In the mMITT set (at least one outcome result), in the PP set (test of cure outcome) and in the modified PP set (missing test of cure rated failure) more than 90% of the patients showed eradication of bacteriuria with nitroxoline, which also met statistical non-inferiority compared to the controls (10% non-inferiority margin) in all three evaluation sets. The clinical efficacy was similar between the two treatment groups.

**Conclusion:**

The IPD meta-analysis using objective parameters (elimination of bacteriuria) demonstrated equivalent efficacy (non-inferiority) of nitroxoline with the controls tested (cotrimoxazole, norfloxacin) in the treatment of uncomplicated UTI. Considering the good safety and efficacy of nitroxoline as also shown in many uncontrolled and observational studies and the world wide increase of resistance of uropathogens against cotrimoxazole and fluoroquinolones, but not against nitroxoline within the last 20 years, nitroxoline should be reconsidered as one of the first line antibiotics for the treatment of uncomplicated UTI.

**Electronic supplementary material:**

The online version of this article (doi:10.1186/s12879-014-0628-7) contains supplementary material, which is available to authorized users.

## Background

Urinary tract infections (UTI) are very frequently found in humans. The most frequent pathogens are *Escherichia coli* and other species of *Enterobacteriaceae*. Gram-positive bacteria, such as staphylococci and enterococci, and non-fermenting Gram-negative bacteria, such as *Pseudomonas aeruginosa*, can also be found [1]–[4]. Funguria due to *Candida spp.* is mainly cultured in patients with risk factors, e.g. immunocompromised or with infections due to foreign bodies [5],[6]. The worldwide increasing bacterial resistance has become a general therapeutic problem. In many countries the resistance rates of *E. coli* against trimethoprim and cotrimoxazole, have exceeded the 20% threshold accepted for empiric therapy by far [1],[3],[7]–[9]. Although in many countries still below 20%, the resistance rates of *E. coli* against fluoroquinolones are also increasing worldwide. Therefore, cotrimoxazole, trimethoprim, fluoroquinolones, the former standard therapeutics for uncomplicated UTI [10], but also amoxicillin with and without clavulanic acid and oral cephalosporins are no more recommended in many of the current guidelines as first line antibiotics for the treatment of uncomplicated UTI (cystitis) [11]–[13]. Older oral antibiotics, such as fosfomycin trometamol, pivmecillinam, and nitrofurantoin, which have still preserved their antibacterial activity against *E. coli* and other uropathogens, are now mostly recommended as first line antibiotics for this indication [11]–[13]. But further alternatives should be looked for.

Nitroxoline, 5-nitro-8-hydroxyquinoline, has been described first in the fifties of the last century [14]. Since the sixties it has been used for treatment and prophylaxis of acute and recurrent UTI [15],[16] in adults and children in the European countries. Nitroxoline is active against most Gram-negative and –positive uropathogenic bacteria [17]–[19], against mycoplasmas (*M. hominis, Ureaplasma urealyticum*) [20],[21] and human pathogenic *Candida spp* [22],[23]. The antibacterial activity against *Acinetobacter spp*., *Enterococcus spp*., and *Serratia spp*. is variable and *Pseudomonas spp*. are considered resistant [17],[19]. Oral treatment with nitroxoline has no qualitative and quantitative effect on the fecal flora [24].

The rate of susceptibility of *E. coli*, the leading uropathogen, at a MIC of nitroxoline of ≤8 mg/l is practically 100% and has not changed over the years as shown by a recent in vitro study with 499 strains from patients with community acquired UTI showing MIC_50_- and MIC_90_-values of 2 mg/l and 4 mg/l, respectively. The unimodal MIC distribution indicates that these pathogens are wild types without any acquired resistance [25]. For other uropathogens, such as *K. pneumonia, P. mirabilis. P. vulgaris, M. morganii, and S. saprophyticus* the MIC_50_- and MIC_90_-values were between 4–8/4–8 mg/l with highest MIC of 8 mg/l [26]. The MIC_90_ for various *Candida spp.*, such as *Candida albicans, Candida tropicalis, Candida parapsilosis, Candida krusei, Torulopsis (Candida) glabrata*, is between 1 and 4 mg/l nitroxoline [27], which correlate well with other studies [28].

The mode of antibacterial and antifungal action is based on the ability of nitroxoline to chelate with various metallic bivalent cations [29]–[31]. The antibacterial actvity of nitroxoline is decreased by the presence of Mn^++^ and Mg^++^, but not Ca^++^ and univalent ions like Na^+^ and K^+^. The authors assume that magnesium ions stabilizing the lipopolysaccharide (LPS) molecules in the outer bacterial membrane favour the accumulation of nitroxoline in the bacteria, such as *E. coli*. This process is energy independent. Nitroxoline is binding to bacterial surface structures, which probably leads to an interaction between nitroxoline and magnesium ions complexing with LPS phosphate groups [30],[31]. The bacterial surface becomes hydrophobic as demonstrated for *E. coli* at 1/8 of MIC of nitroxoline and the adherence to catheter surface is decreased [32]. Nitroxoline inhibits rapidly and selectively RNA synthesis in fission yeast by chelation of bivalent cations required for RNA synthesis [33]. Furthermore, subinhibitory concentrations of nitroxoline have a major inhibitory effect on adhesin expression and bacterial attachment [34]–[36], which may add an important antibacterial mechanism of nitroxoline. Nitroxoline also reduces the formation and induces the dispersal of Pseudomonas aeruginosa biofilms by chelation of iron and zinc [37].

After single oral administration of 200 mg nitroxoline antibacterial urinary activity can be demonstrated by microbiological assays peaking on average between 216 mg/l and 220 mg/l during the urine collection periods 1–2 hours and 3–4 hours [38]. The HPLC concentrations of free nitroxoline, however, were much lower peaking on average between 3.2 mg/l and 5.8 mg/l during that periods. The discrepancy between the two assays was explained by antibacterial active metabolites in urine, because nitroxoline is excreted in the urine in unconjugated as well as conjugated (glucuronide, sulfate etc.) forms [38],[39]. A recent study showed that after oral administration of 250 mg about 99% of the excreted nitroxoline was eliminated as metabolites in urine of volunteers, mainly as sulfate and glucuronide, and only 1.0% as the parent component [40],[41]. In this study urinary inhibitory and bactericidal concentrations were determined against selected uropathogens, such as *E. coli, K. pneumonia, P. mirabilis, and S. saprophyticus.* In connection with additional urinary bactericidal kinetic studies it was concluded, that nitroxoline exhibits mainly bacteriostatic activity in urine against susceptible strains. Its antibacterial activity is more pronounced in acidic than in alkaline urine.

The aim of this article is to review systematically the available literature on the use of nitroxoline for treatment and prevention of UTI and to meta-analyse four prospective, randomized, single-blind, multicenter, comparative studies, so far unpublished, in order to improve the evidence based recommendation to use nitroxoline as one of the first line drugs for the treatment of uncomplicated UTI (cystitis).

## Methods

### Literature search

A narrative literature search concerning efficacy and safety of nitroxoline in the treatment of UTI was performed by the manufacturing company (Rosen Pharma, Blieskastel, Germany). A total of 26 uncontrolled studies including 1206 patients (947 adults and 259 children) published between 1962 and 1987, two controlled studies including 148 patients (100 adult and 48 children) and one postmarketing observational study [42] including 9800 patients with uncomplicated and complicated UTI were found (Tables 1, 2, 3 and 4).

**Table 1:** **Total number (n) of published clinical studies on treatment of urogenital infections with nitroxoline as found by the narrative literature search**

Study design	Population	Studies (n)	Patients (n)
Uncontrolled	Adults	15	947
Uncontrolled	Children	11	259
Controlled vs. norfloxacin	Adults	1	100
Controlled vs. TMP/SMX	Children	1	48
Observational	Adults	1	9,800
**Total**		**29**	**11,154**

TMP/SMX – trimethoprim/sulfamethoxazole.

**Table 2:** **Fifteen uncontrolled clinical studies with nitroxoline in 947 adult patients of both genders**

First author	Year	Pat (n)	Indication (T/P)	Dosage	Duration	Success rate	Adverse events
Kuss [43]	1962	72	T: acute cUTI & uUTI	400 mg/d	20–45 days	78%	1.3% gastrointestinal
Moreau [44]	1962	20	T: acute cUTI	400–500 mg/d	8–10 (−45) days	90%	5% gastrointestinal
v. Rütte [45]	1969	200	P: chronic rUTI	300–500 mg/d	2–3 months	80%	0%
				shortly 800 mg/d	1 day		
Uhlir [46]	1972	20	T: acute UTI (7), chronic PN (13)	300 mg/d	14 days	100%	0%
Allal [47]	1973	264	T: UTI during pregnancy	300 mg/d	6 days	> 75%	No data
Bittard [48]	1974	50	P: post-op. catheter	7.5–10 mg/kg/d	6 weeks	92%	Few gastrointestinal
Schlesinger [49]	1975	65	T: chronic PN (62), chronic prostatitis (3)	300–500 mg/d	10 days	80% clinical	0%
Aubert [50]	1976	28	T: post-op. catheter, after endoscopy	200–300 mg/d	10–15 days	72%	0%
Dufour [51]	1979	15	T: acute prostatitis	900–1600 mg/d	3–5 days	81%	No data
Lenzner [52]	1983	60	T: fungal UTI	750 mg/d	10–20 days	80%	3.3% itching; few cases with nausea and vomiting
Schülke [53]	1984	50	T: postop., acute UTI after removal of urethral catheter for 3–10 days	750 mg/d	3 days	78%	0%
Sachse [54]	1984	44	P: chronic rUTI	750 mg/d	4 months	77% free of rUTI; rUTI rate decreased from 0.33 to 0.11/month	9% gastrointestinal 2.2% exanthema
Demontrond [55]	1986	15	T: candiduria in hospitalised patients	600 mg/d	10–30 days	87%	0%
Frobert [56]	1987	36	T: acute, uUTI in hospitalised patients	600 mg/d	10 days	93% bacteriological	5.5% gastrointestinal
						87% clinical	2.7% nausea
							2.7% dizziness
Cancet [27]	1987	8	T: urogenital fungal infections	600 mg/d	15 days	100%	no data

T-therapy; P-prophylaxis; UTI-urinary tract infection; uUTI-uncomplicated UTI; cUTI-complicated UTI; PN-pyelonephritis.

**Table 3:** **Eleven uncontrolled clinical studies on treatment and prophylaxis of UTI with nitroxoline in 259 children**

First author	Year	Patients (n)	Indication (T/P)	Dosage	Duration	Success rate	Adverse events
Lecornu [57]	1974	25 children	T: compl. and uncompl. UTI	50–400 mg/d as suspension	4–12 days	72%	8% nausea
		0–13 years					
Roussel [58]	1974	24 children	T: compl. and uncompl. UTI	10 mg/kg/d as suspension	10 days- 6 months	79%	0%
		7 days–6 months					
Raynaud [59]	1974	19 children	T: compl. and uncompl. UTI	10 mg/kg/d as suspension	20 days	69%	10% nausea
		0–10 years					
Luckel [60]	1975	25 children	P: compl. and uncompl. UTI	10–20 mg/kg/d as suspension	17–55 days	83%	0%
		0–8.5 years					
Viville [61]	1975	22 children	P: compl. UTI	10–30 mg/kg/d as suspension	3–6 months	86%	0%
		2 months-17 years					
Chable [62]	1975	28 children	T: compl. and uncompl. UTI	10–20 mg/kg/d as suspension	10 days	81%	0%
		2 month-14.5 years					
Battin [63]	1975	30 children	P: compl. and uncompl. UTI	10 mg/kg/d as suspension	6 weeks	90%	0%
		2 month-10 years					
Sorez [64]	1975	30 children	T: compl. and uncompl. UTI	10–25 mg/kg/d as suspension	10 – 17 days	73.7% uncompl. UTI 40% compl. UTI	0%
		10 days-8 years					
Neimann [65]	1975	21 children	T/P: compl. and uncompl. UTI	25–400 mg/d as suspension	4 days-4 months	90%	10% nausea
		26 days-8 years					
Machecourt [66]	1976	23 children	T: compl. and uncompl. UTI	10–20 mg/kg/d as suspension	10 days	91%	0%
		21 days-14 years					
Lambert-Zechovsky [24]	1987	12 children	T: uncompl. UTI	20 mg/kg/d as suspension	10 days	66% (91% incl. non-compliance)	-
		aver. 4 years					

n-number; T-therapy; P-prophylaxis; UTI-urinary tract infection.

**Table 4:** **Two controlled open clinical studies in a total of 148 patients with nitroxoline (NTX) versus norfloxacin (NFX) or cotrimoxazole (CTX); SMX-sulphamethoxazole; TMP-trimethoprim**

First author	Year	Pat.(n)	Indication (T/P)	Antibiotic and dosage	Duration	Success rate	Adverse events
Schülke [67]	1986	51 NTX	T: postop., uncompl. UTI	750 mg NTX/d vs. 800 mg NFX/d	3 days	60.8% NTX 59.2% NFX	0%
		49 NFX					
Dodat [68]	1988	48 children	P: postop. UTI (ureteral reflux)	10 mg NTX/kg/d	30–60 days	95% NTX	6% NTX
		0–8 years		vs. 15 mg SMX/ 3 mg TMP/kg/d		95% CTX	5% CTX

T-therapy; P-prophylaxis, UTI-urinary tract infection.

### IPD Meta-analysis of four clinical studies

The individual patients’ data (IPD) of four prospective, randomized, single-blind, multicenter, comparative studies (data on file, Rosen Pharma GmbH), so far unpublished, were available for this IPD meta-analysis. The four studies were performed to renew marketing registration during December 1992 until 1993 in 20 investigational sites. A total of 466 female patients suffering from acute sporadic cystitis or acute episode of recurrent cystitis was included in the four studies (nitroxoline: n = 234; norfloxacin: n = 54; cotrimoxazole: n = 178). For the meta-analysis the original individual patient’s data (IPD) could be analyzed.

The studies were performed in accordance with the declaration of Helsinki updated in Hongkong 1989 [69]. Before start they were voted positively by the Ethical Commissions of the Medical Councils of Saarland for study NWNF #10 (dated 7 Sept 1992), Bavaria for study NWNF #11 (dated 20 October 1992, Nr. 92247), Rheinland-Pfalz for study NWNF #13 (dated 3 November 1992), and Hessen for study NWNF # 15 (dated 2 November 1992, Nr. 71/92), and the respective countries of Germany where the studies where performed. A registration in a public trials registry was not required at that time.

#### Study design

The four clinical studies were prospective, randomized, single-blind (patient blinded), multicenter, and comparative in parallel groups. The aim of the studies was to compare the efficacy and safety of nitroxoline versus cotrimoxazole and norfloxacin in female patients with acute uncomplicated sporadic cystitis (NWNF 10, 11, 13) or with acute episode of recurrent uncomplicated cystitis (NWNF 15). In Table 5 the main parameters of the design of the four studies are outlined.

**Table 5:** **Study design of the four meta-analysed prospective, open, randomised clinical studies in female patients with acute uncomplicated and recurrent cystitis treated with nitroxoline (NTX) versus a control antibiotic, cotrimoxazole (CTX) or norfloxacin (NFX)**

Study	Nitroxoline	Control	Indication	Patients (n)	Duration	End of therapy visit	Test of cure visit
NWNF 10°	Nitroxoline (NTX)	Cotrimoxazole (CTX)	Acute uncompl. cystitis	130 total	5 days	day 6	day 12–14
	3×250 mg	2×960 mg		67 NTX, 63 CTX			
NWNF 11	Nitroxoline (NTX)	Cotrimoxazole (CTX)	Acute uncompl. cystitis	115 total	5 days	day 6	day 12–14
	3×250 mg	2×960 mg		56 NTX			
				59 CTX			
NWNF 13	Nitroxoline (NTX)	Norfloxacin (NFX)	Acute uncompl. cystitis	105 total	5 days	day 6	day 12–14
	3×250 mg	2×400 mg		51 NTX			
				54 NFX			
NWNF 15	Nitroxoline (NTX)	Cotrimoxazole (CTX)	Acute episode of uncompl. recurrent cystitis	116 total	10 days	day 11–13*	day 21–23
	3×250 mg	2×960 mg		60 NTX			
				56 CTX			

°inclusion criteria missing (no urine cultures reported): total 12, NTX 7, CTX 5 patients (included in safety set only).

*additional visit during therapy: day 6–8.

#### Selection of patients

After medical history including kind and duration of symptoms, pretreatment and concurrent medication, physical examination including body temperature, urinalysis and confirmation of the clinical diagnosis including additional tests needed, eligible patients were informed about aim, performance, possible adverse events and their right to terminate the study without giving any reasons. If written informed consent was documented, patients were included into the study after inclusion and exclusion criteria had been checked.

In three studies (NWNF 10, 11, 13) female patients with clinical signs and symptoms of acute sporadic uncomplicated cystitis and in one study (NWNF 15) those with acute episode of recurrent uncomplicated cystitis were included. In all patients a pretreatment midstream or single catheter urine sample was requested to show pyuria (white blood cells >5 000/ml) and bacteriuria with colony forming units (CFU) ≥ 10^5^/ml due to susceptible pathogens.

According to the study protocol exclusion criteria were: age below 18 years; taking part in a clinical study during the last 30 days; pregnancy; urolithiasis or obstruction within the urinary tract; severe systemic, gastrointestinal, kidney or liver diseases; hypertension; diabetes mellitus; known erythema exsudativum multiforma, pathologic hemogram including hemoglobin anomalies; glucose-6-phosphate-deficiency; acute porphyria; folic acid deficiency; known seizure disorder; and allergy against sulfonyl urea antidiabetics and sulfonamide based diuretics.

#### Randomization, treatment, and visits

Female patients were randomized in two age groups (upto 45 and above 45 years) and received their allocated antibiotic wrapped in a neutral box signed “for clinical study only”. Because an objective outcome (eradication of bacteriuria) was the primary aim of the study, a double-blind design was not considered necessary at that time.

Patients in the nitroxoline group were asked to take one capsule containing 250 mg nitroxoline three times daily with the three main meals. Patients of the control group were asked to take one tablet containing 960 mg cotrimoxazole (NWNF 10, 11, 15) or 400 mg norfloxacin (NWNF 13) twice daily with the morning and evening meal. The treatment duration was 5 days in 3 studies (NWNF 10, 11, 13) and 10 days in one study (NWNF 15). The control visits were one day (end of treatment) and 7–13 days (test of cure) after end of therapy. In the ten day treatment study (NWNF 15) an additional visit during therapy at day 6 was scheduled.

##### Primary objectives

The primary objective was the eradication (CFU <10^4^/ml) of the bacteriuria due to susceptible pathogens at test of cure in compliant patients (≥80% of medication) without taking any additional antibiotic.

##### Secondary objectives

Secondary objectives were clinical efficacy and safety. For clinical efficacy the following symptoms were scored (0-absent upto 5-very severe) by the patient: dysuria, frequency, urgency, nycturia, flank/back pain. The clinical efficacy was assessed also globally by the patient and the investigator. At each visit patients were asked for any adverse event, which was documented. The safety was assessed also globally by the patient and the investigator.

The following laboratory tests were performed: Urinalysis (white and red blood cells, nitrite reaction, protein, glucose, pH), serum chemistry (glucose, creatinine, bilirubin, AST, ALT, gammaGT, sodium, potassium, calcium) and hematology (red and white blood count, thrombocytes, blood sedimentation rate).

#### Evaluation sets

The efficacy and safety was analysed in the following patients’ sets:

***Safety set:*** All patients included into the study having received any study medication.

***Modified microbiologically Intention-to-Treat Set (mMITT):*** All patients with significant bacteriuria (CFU ≥10^5^/ml) at study entry; with at least one control visit including microbiological investigation, and with a consistent clinical report (in study NWNF 10 few clinical reports were inconsistent). The last observation was brought forward (LOF), if not failure has already been documented and an additional antibiotic been administered.

***Per Protocol Set (PP):*** All patients with significant bacteriuria (CFU ≥10^5^/ml) at study entry; with test of cure visit including microbiological investigation (except failure was already documented and additional antibiotic administered); doubtless randomization; and compliance (≥80% of allocated drug).

***Modified per Protocol Set (mPP):*** All patients with significant bacteriuria (CFU ≥10^5^/ml) at study entry; with test of cure visit including microbiological investigation; missing test of cure visit was rated as failure (worst case) [70]; doubtless randomization; and compliance (≥80% of allocated drug).

#### Statiscial analysis

The individual patient data were entered and independently checked to ensure high data quality. Categorial data are described by absolute and relative frequencies. Ordinal data are either described in tables by absolute and relative frequencies (global clinical assessment) or by box plots (scoring of clinical symptoms). For numerical data median as well as minimum and maximum value are given.

To evaluate the noninferiority of nitroxoline compared to the controls with regard to the failure rate the difference of the failure rates of the two treatments and its 95%-confidence interval is estimated [71]. Odds Ratios are estimated, too. Difference as well as odds ratio are estimated by logistic regression with random center effect [72]. Since the proportion of variance explained by the centers exceeded substantially the proportion of variance explained by the four studies random center effects instead of random study effects were added to the model. Wether the distribution of ordinal data is the same in the two treatment groups is analyzed by ordered probit regression, the distribution of numerical data is compared by linear regression. For both approaches random center effects are taken into account.

All p-values are two-sided, the level of significance is set to 0.05. Stata/IC 13.1 was used for data preparation and statisical analysis.

## Results

### Literature search

The 26 uncontrolled clinical studies published between 1962 and 1987 included a total of 1206 patients (947 adults and 259 children) (Table 1). Nitroxoline was mainly administered for treatment of uncomplicated and complicated UTI as well as for prophylaxis of recurrent UTI with daily dosages mostly between 300 and 900 mg. The treatment duration varied mainly between 3 and 10 days depending on the indication. Study details are presented in Tables 2 and 3.

The summary results of the two controlled studies including 148 patients (100 adult and 48 children) are outlined in Table 4. In one study 51 patients received 250 mg nitroxoline tid versus 49 patients 400 mg norfloxacin bid for treatment of postoperative and uncomplicated UTI [67]. The microbiological outcome was comparable in both groups (about 60%); no adverse events were reported. The second controlled study was performed in 48 children with ureteral reflux [68]. The children received either nitroxoline or cotrimoxazole for prophylaxis of UTI for 30–60 days. In 95% of the children a recurrent UTI could be prevented in both groups. Adverse reactions were reported in 6% with nitroxoline and in 5% with cotrimoxazole.

The postmarketing observational study included 9800 patients (75% women, 25% men) with complicated and uncomplicated UTI including recurrent UTI, and to a lesser extent also with fungal urogenital infections [42]. The dosage of nitroxoline was fixed at 250 mg tid, but the treatment duration was chosen according to the physician’s decision. The physician’s assessment revealed clinical success of 95% in cystitis, 79% in recurrent and complicated UTI; 80% in pyelonephritis, and 91% in fungal infections. The overall patient’s assessment of success was 95%. Only 2.2% of the patients reported adverse events overall including 1.8% gastrointestinal and 0.3% allergic adverse events.

### IPD meta-analysis

#### Patients

The studies were performed in 20 German cites from Dezember 1992 until 1993. A total of 466 female patients (nitroxoline: n = 234, controls: norfloxacin: n = 54; cotrimozazole: n = 178) was included in the four clinical studies. In the IPD meta-analysis the results obtained from patients in the nitroxoline group were compared with those obtained from patients in the control groups, norfloxacin and cotrimoxazole.

#### Demography, history and physical examination

In Table 6 some data concerning demography, history and physical examination are compared between nitroxoline and controls in the safety set. A significant difference was only found as a slightly higher body mass index, higher systolic blood pressure and a lower proportion pretreated by antibiotics in the group of the controls. Both groups included about the same number of patients upto 45 and above 45 years. Although diabetes mellitus and hypertension, considered as exclusion criteria in the original protocol, was found in some patients in both groups, these patients were not excluded from the meta-analysis, because these underlying diseases were considered only minor protocol violations.

**Table 6:** **Demography, history, and physical parameters of female patients investigated in the safety set**

	Nitroxoline		Controls		p-value
Demographic parameter	n	Median (range)	n	Median (range)	
Age (years)	234	48 (18–89)	232	47 (18–85)	0.520
-up to 45 years	234	113 (48.3%)	232	115 (49.6%)	0.745
Height (cm)	234	165 (150–185)	231	165 (145–187)	0.290
Weight (kg)	234	62 (43–100)	232	64 (42–102)	0.125
Body mass index (kg /m^2^)	234	23.0 (15.9–41.3)	231	23.2 (15.6–38.0)	0.038
Heart rate (min ^−1^)	231	80 (52–172)	231	80 (56–120)	0.354
Systolic BP (mmHg)	232	120 (80–195)	231	130 (100–190)	0.025
Diastolic BP (mmHg)	232	80 (60–120)	231	80 (50–120)	0.410
No hypertension (SBP ≤ 140 mmHG)	232	165 (71.1%)	231	137 (59.3%)	0.004
Mild hypertension (SBP > 140 mmHG)	232	46 (19.8%)	231	63 (27.3%)	
Moderate hypertension (SBP > 160 mmHG)	232	17 (7.3%)	231	22 (9.5%)	
Severe hypertension (SBP > 180 mmHG)	232	4 (1.7%)	231	9 (3.9%)	
Diabetes mellitus	234	11 (4.7%)	232	6 (2.6%)	0.230
Duration of urinary symptoms (d)	165	3 (1–30)	168	3 (1–21)	0.442
Pretreatment with antimicrobials*	234	16 (6.8%)	232	8 (3.4%)	0.060
Only unspecific pretreatment^#^	234	25 (10.7%)	232	28 (12.1%)	0.667

*antibiotics, chemotherapeutics.

^#^spasmolytics, analgetics, tea etc.

#### Flow of patients

**Figure 1:**
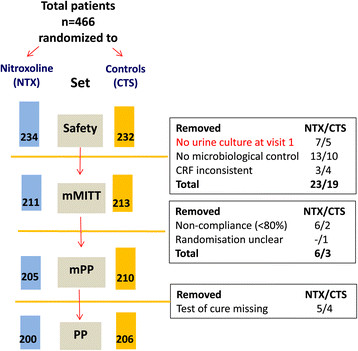
**Diagram showing flow of patients participating in the four studies on treatment with nitroxoline or with a contol antibiotic, cotrimoxazole or norfloxacin.** mMITT-modified microbiological intention to treat; mPP-modified per protocol; PP-per protocol.

In Figure 1 the total number of patients included in the studies is shown, randomized to nitroxoline and controls and excluded because of different reasons from the evaluation sets. Finally the safety (mMITT/mPP/PP) set comprised 234 (211/205/200) patients in the nitroxoline and 232 (213/210/206) in the control groups.

#### Quality of the studies

Each of the four randomized clinical trials achieved a good quality with four out of maximum five Jadad scores [73],[74]: randomization (1 score), which was appropriate (1 score), patient blinding only (0 score), which was appropriate (1 score), and drop-outs well-grounded (1 score).

#### Bacterial spectrum and resistance to study drugs

The bacterial spectrum and resistance at study entry (visit 1) is shown in Table 7. On average about 10% of the patients were included with mixed infections. Therefore the total number of isolates were higher than the number of patients included. *Escherichia coli* was the most frequent pathogen in all studies ranging from 65% to 73% of all isolates. The percentages of resistant isolated to nitroxoline ranged from 0% to 5.5% and that to the control antibiotics from 0.8% to 5.2%. There was no evidence, that patients with recurrent UTI (study NWNF 15) showed more resistant strains than patients with sporadic UTI (studies NWNF 10,11,13).

**Table 7:** **Bacterial spectrum and resistance (R) against nitroxoline (Ni) versus controls (C), cotrimoxazole or norfloxacin, at study entry (visit 1)**

Study Nr./R	NWNF 10	R(n)	NWNF 11	R(n)	NWNF 13	R(n)	NWNF 15	R(n)	NWNF 10–13	R(n)	NWNF 10–15	R(n)
Bacterial isolates	n (%)	Ni/C	n (%)	Ni/C	n (%)	Ni/C	n (%)	Ni/C	n (%)	Ni/C	n (%)	Ni/C
*E. coli*	82(67.2%)	-/1	88(65.3%)	1/6	81(67.0%)	−/−	98(73.0%)	−/−	251(66.4%)	1/7	349(68.2%)	1/7
*Proteus spp*.*	20(16.4%)	−/−	9(6.7%)	−/−	8(6.6%)	−/−	12(9.0%)	−/−	37(9.8%)	−/−	49(9.6%)	−/−
*Klebsiella spp*.	1(0.8%)	−/−	5(3.7%)	−/−	1(0.8%)	−/−	6(4.5%)	−/−	7(1.9%)	−/−	13(2.5%)	−/−
*Enterobacter spp*.	-	-	2(1.5%)	−/−	3(2.5%)	−/−	7(5.2%)	-/2	5(1.3%)	−/−	12(2.3%)	-/2
*Citrobacter spp*.	2(1.6%)	−/−	-	−/−	1(0.8%)	−/−	1(0.8%)	−/−	3(0.8%)	−/−	4(0.8%)	−/−
Enterococci	9(7.4%)	−/−	13(9.7%)	6/1	13(10.7%)	-/1	2(1.5%)	−/−	35(9.3%)	6/2	37(7.2%)	6/2
Staphylococci	5(4.1%)	−/−	7(5.2%)	−/−	13(10.7%)	1/-	8(6.0%)	−/−	25(6.6%)	1/-	33(6.4%)	1/-
Streptococci	2(1.6%)	−/−	2(1.5%)	−/−	1(0.8%)	−/−	-	−/−	5(1.3%)	−/−	5(1.0 %)	−/−
Others	1(0.8%)	−/−	9(6.7%)	−/−	-	−/−	-	−/−	10(2.6%)	−/−	10(2.0%)	−/−
**Total**	122(100%)	-/1	135(100%)	7/7	121(100%)	1/1	134(100%)	-/2	378(100%)	8/9	512(100%)	8/11
		-/0.8%		5.2/5.2%		0.8/0.8%		-/1.5%		2.1/2.4%		1.6/2.1%
Isolates/patients	122/122		135/115		121/105		134/116		378/350		512/466	
	(1.0)		(1.17)		(1.15)		(1.16)		(1.08)		(1.1)	

*indol positive and negative strains.

#### Microbiological outcome

The micobiological outcome (eradication of bacteriuria <10^4^/ml) as primary study aim is shown in Table 8. In all three evaluation sets the eradication rates were above 90% in the nitroxoline and in the control groups. The 95% confidence intervals (CI) of the estimated eradication rate differences met statistical non-inferiority compared to the controls (10% non-inferiority margin) in all three evaluation sets.

**Table 8:** **Microbiological success (eradication of bacteriuria) in the different evaluation sets**

Evaluation set	Nitroxoline	Controls	Difference (%) (95%-CI)	Odds ratio (95%-CI)
mMITT	192/211 (91.0%)	203/213 (95.3%)	−3.4 (−9.7 to 3.0)	0.47 (0.21 to 1.06)
PP	184/200 (92.0%)	197/206 (95.6%)	−2.2 (−8.2 to 3.7)	0.47 (0.19 to 1.14)
mPP	185/205 (90.2%)	197/210 (93.8%)	−2.9 (−9.9 to 4.1)	0.60 (0.28 to 1.27)

mMITT-modified microbiological intention to treat; PP-per protocol; mPP-modified per protocol.

Difference: nitroxoline – controls.

Odds Ratio: nitroxoline vs controls.

Estimation by logistic regression with random center effect.

**Figure 2:**
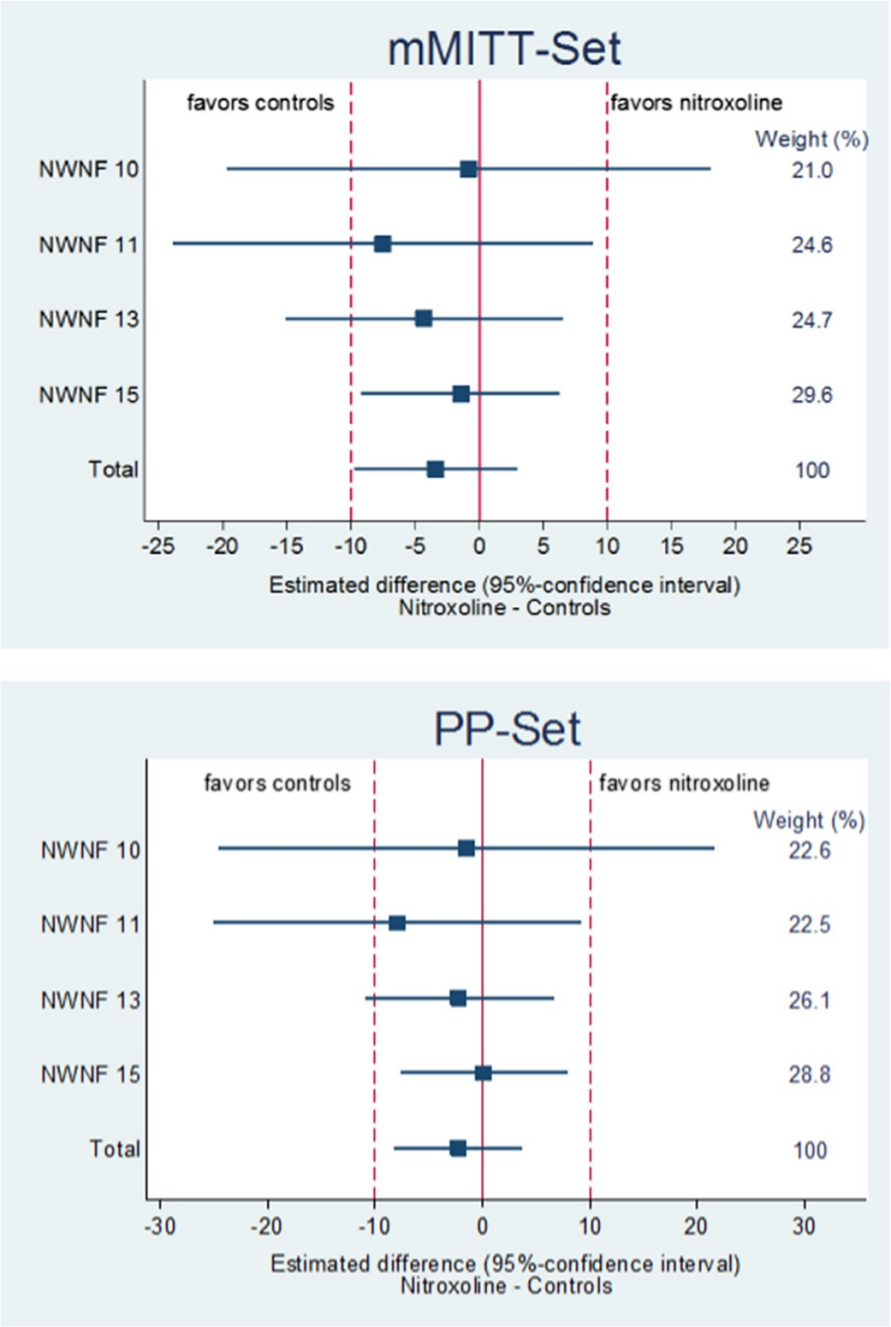
**Estimated difference (95%-confidence interval) between the microbiological success and weights from random effects analysis in the mMITT-Set and PP-Set of patients treated with nitroxoline as compared to controls in the four studies analysed.** mMITT-modified microbiological intention to treat; PP-per protocol.

Figure 2 shows the 95% CI of the individual studies and the total result of the four meta-analysed studies together in the mMITT and the PP sets with the contributing weight of each study.

The eradication rates of *E. coli* in mono and mixed infection and Non-*E. coli* mono and mixed infections in the PP set are shown in Table 9. As expected about 80% (79.0% vs 79.6%) of the patients in both groups had an *E. coli* infection either as mono or mixed infection with overall eradication rates of 94.3% and 96.3%, respectively. In the about 20% Non-*E. coli* infections the overall eradication was 83.3% and 93.0% in the nitroxoline and the control groups, respectively.

**Table 9:** **Microbiological success (eradication of bacteriuria) according to bacterial groups in the PP set**

	Nitroxoline	Controls
Monoinfection *E. coli*	129/138 (93.5%)	141/145 (97.2%)
Mixed infection *E. coli*	20/20 (100%)	17/19 (89.5%)
Monoinfection with Non-*E. coli*	28/34 (82.4%)	37/40 (92.5%)
Mixed infection with Non-*E. coli*	7/8 (87.5%)	2/2 (100%)
Total	184/200 (92.0%)	197/206 (95.6%)

#### Clinical efficacy

**Figure 3:**
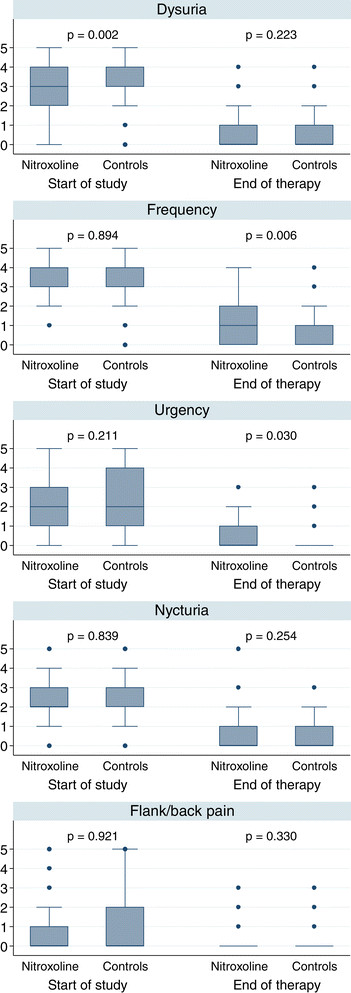
**Clinical efficacy (symptom scoring) in the PP treated by nitroxoline (n=193) and the controls (n= 203) (no assessment in 10 patients).**

In Figure 3 the clinical efficacy in the PP sets are presented as reduction of symptoms as scored pre- and posttreatment. At the end of therapy no significant differences were seen in dysuria (pain during micturition), nocturia and flank/back pain between the two groups. Marked reductions of frequency (pollakisuria) and urgency (imperative micturition) were also seen (included) in both groups, which were, however, more pronounced in the controls. Considering the overall low scores achieved at the end of therapy, the differences may not be clinical relevant.

#### Global clinical assessment

In the global clinical assessment (Table 10) the patient (investigator) rated the treatment with nitroxoline in 91.2% (90.5%) and that with the control antibiotics in 96.2% (95.5%) as very good or good.

**Table 10:** **Global clinical assessment by the investigator in the PP set (no assessment in 103 patients)**

Assessment	Investigator		Patient	
Group	Nitroxoline (n = 148)	Controls (n = 158)	Nitroxoline (n = 148)	Controls (n = 158)
Very good	115 (77.7%)	138 (87.3%)	109 (73.6%)	123 (77.8%)
Good	19 (12.8%)	13 (8.2%)	26 (17.6%)	29 (18.4%)
Satisfactory	4 (2.7%)	5 (3.2%)	5 (3.4%)	4 (2.5%)
Moderate	1 (0.7%)	0 (0%)	3 (2.0%)	1 (0.6%)
Insufficient	9 (6.1%)	2 (1.3%)	5 (3.4%)	1 (0.6%)
p-value*	0.006		0.143	

*comparison of nitroxoline vs. controls (ordered probit regression with random center effect).

#### Safety

The treatments with nitroxoline and the control antibiotics were generally well tolerated (Table 11). A total of 9.8% treated with nitroxoline and 7.8% treated with a control antibiotic reported any adverse event (p = 0.360). Only 5 (nitroxoline 3; controls 2) patients discontinued the treatment prematurely because of adverse events. The main adverse events were gastrointestinal disorders (nitroxoline 19; controls 9). In 3 patients of each group allergic reactions including itching, urticaria and in one case with papules/pustules (nitroxoline) were seen.

**Table 11:** **Adverse events**

Adverse events (AE)		Nitroxoline	Controls
	Patients (total)	234 (100%)	232 (100%)
	Patients with AEs^#^	23 (9.8%)	18 (7.8%)
Adverse events (total)		33	26
Nervous system disorders	Headache	3	4
	Dizziness	-	3
Eye disorders	Eyelid swelling	1	-
Cardiac and circulation disorders	Circulation disorder	-	1
Gastrointestinal disorders	Dysgeusia	-	1
	Nausea	9	3
	Regurgitation	1	1
	Gastralgia	4	3
	Obstipation	-	1
	Diarrhoea	5	-
Metabolim and nutrition disorder	Anorexia	1	-
Urinary disorders	Incontinence	1	-
Reproductive system disorders	Colpitis	1	1
	Fluor vaginalis	1	-
	Tubal tumor	-	1
Immune system disorders	Allergy	1*	1
Skin and subcutaneous tissue disorders	Itching	1	1
	Urticaria	1	1
	Herpes labialis	-	1
General disorders	Intolerance	1	-
	Weakness	1	-
	Fever	1	-
Musculoskeletal, connective tissue and bone disorders	Back pain	-	1
	Flank pain	-	2
	Leg pain	-	1

^#^comparison of nitroxoline vs. controls (logistic regression with random center effect) p = 0.360; *with papules/pustules.

In the laboratory parameters (Table 12) no significant differences between critical values were observed between the two treatment groups (nitroxoline/controls), except a higher pretreatment (12.8% vs. 6.4%), but not posttreatment rate of critical values regarding the number of blood erythrocytes in the nitroxoline group.

**Table 12:** **Laboratory parameters before and after treatment in the safety-set**

	Critical values	Pretreatment			Posttreatment		
		Nitroxoline	Contols	p-value*	Nitroxoline	Contols	p-value*
Glucose (mg/dl)	< 55, >160	20/227 (8.8%)	12/226 (5.3%)	0.138	12/206 (5.8%)	7/207 (3.4%)	0.242
Creatinine (mg/dl)	> 1.8	1/228 (0.4%)	1/227 (0.4%)	0.998	1/205 (0.5%)	0/207 (0%)	-
Bilirubin (mg/dl)	< 0.1, >2.0	3/226 (1.3%)	1/227 (0.4%)	0.337	2/205 (1.0%)	4/207 (1.9%)	0.426
SGOT –AST (U/l)	< 35, > = 150	224/226 (99.1%)	225/226 (99.6%)	0.570	203/205 (99.0%)	206/207 (99.5%)	0.564
SGPT - ALT (U/l)	< 35, > = 150	222/226 (98.2%)	225/226 (99.6%)	0.212	204/205 (99.5%)	207/207 (100%)	-
gamma-GT (U/l)	< 3, >38	9/227 (4.0%)	10/226 (4.4%)	0.807	9/205 (4.4%)	6/207 (2.9%)	0.412
Sodium (mmol/l)	< 130, >150	8/228 (3.5%)	6/226 (2.7%)	0.603	11/205 (5.4%)	5/207 (2.4%)	0.132
Potassium (mmol/l)	< 3.6, >6.0	22/228 (9.6%)	19/226 (8.4%)	0.628	19/205 (9.3%)	12/206 (5.8%)	0.229
Calcium (mmol/l)	< 8, >11.5	229/229 (100%)	227/227 (100%)	-	206/206 (100%)	206/206 (100%)	-
Hemoglobin (g/dl)	< 10, >17	0/227 (0%)	0/219 (0%)	-	1/201 (0.5%)	2/204 (1.0%)	0.582
Hematocrit (%)	< 32, >47	25/227 (11.0%)	33/220 (15.0%)	0.197	20/201 (10.0%)	33/204 (16.2%)	0.065
Erythrocytes (Mio/μl)	< 4, >5.1	29/227 (12.8%)	14/220 (6.4%)	0.024	22/201 (10.9%)	26/205 (12.7%)	0.588
Leucocytes (Tsd/μl)	< 4, >11.3	25/227 (11.0%)	21/220 (9.5%)	0.605	13/200 (6.5%)	16/204 (7.8%)	0.614
Neutrophiles (%)	< 39, >75	22/225 (9.8%)	37/221 (16.7%)	0.031	15/199 (7.5%)	13/203 (6.4%)	0.656
Eosinophiles (%)	< 1, >10	10/223 (4.5%)	19/219 (8.7%)	0.080	11/199 (5.5%)	8/203 (3.9%)	0.399
Basophiles (%)	0, >15	38/222 (17.1%)	47/220 (21.4%)	0.257	29/199 (14.6%)	38/203 (18.7%)	0.266
Lymphocytes (%)	< 10, >50	9/221 (4.1%)	12/220 (5.5%)	0.391	7/198 (3.5%)	11/203 (5.4%)	0.382
Monocytes (%)	< 3, >25	7/223 (3.1%)	14/220 (6.4%)	0.117	9/199 (4.5%)	6/203 (3.0%)	0.410
Retikulocytes (%)	< 0.5, >2	220/224 (98.2%)	212/218 (97.2%)	0.493	198/200 (99.0%)	200/205 (97.6%)	0.227
Thrombocytes (Tsd/μl)	< 150, >450	11/225 (4.9%)	8/218 (3.7%)	0.529	10/201 (5.0%)	8/204 (3.9%)	0.608
BSR (mm/1 h)	< 5, >11	134/203 (66.0%)	124/202 (61.4%)	0.343	106/179 (59.2%)	114/187 (61.0%)	0.756
BSR (mm/2 h)	> 25	82/202 (40.6%)	88/200 (44.0%)	0.470	44/178 (24.7%)	57/186 (30.6%)	0.207

BSR-blood sedimentation rate.

*comparison of nitroxoline vs. controls, logistic regression with random center effect.

## Discussion

When nitroxoline was first marketed in the European Union in 1962 for treatment and prophylaxis of UTI, microbiological and observational studies were considered sufficient. For this reason between 1962 and 1987 only uncontrolled studies were published not only for treatment and prophylaxis of uncomplicated, but also for treatment of complicated UTI including pyelonephritis, prostatitis and fungal infections in adults of both genders, and also in children. Only two prospective randomized studies, one for treatment of uncomplicated UTI in adults and one for prophylaxis of UTI in children, were published, both with low number of patients [67],[68]. Although these studies may not be considered highly evidential, they can at least contribute to establish the safety of nitroxoline in adults as well as in children together with the large postmarketing observational study.

When the requirements became more stringent for the renewal of the regulatory approval, finally achieved in Germany in 2005, four prospective randomized single blind studies were performed during 1992 and 1993 with cotrimoxazole and norfloxacin as controls. All four studies followed in essence the same protocol with the only difference that the treatment duration in women with sporadic uncomplicated UTI was five days and in women with acute episode of recurrent UTI was ten days. All four studies were of high quality achieving four out of maximum five Jadad scores [73],[74]. The bacterial spectra were similar between the studies with *E. coli* as most frequent pathogen ranging from 65% to 73%. There was no evidence that females suffering from recurrent UTI showed more resistant strains than those with sporadic UTI. During the years 1992–1993, when the studies were performed, the resistance rates for cotrimoxazole and norfloxacin were still very low. Both antibiotics were considered first line drugs for the treatment of uncomplicated UTI at that time [10]. For all these reasons, including the data of all four studies in one meta-analysis was justified.

Since the clinical report forms (CRF) of the individual patients were available, an IPD meta-analysis could be performed, a specific type of systematic review [75]. Rather than extracting summary (aggregate) data from study publications, the original research data are sought directly for each study. These data can then be re-analysed centrally and combined, if appropriate, in meta-analyses. IPD meta-analyses can improve the quality of data and their analysis and thus produce more reliable results [76]. For this reason they are considered a ‘gold standard’ of systematic review in contrast to meta-analyses of aggregate data, which relates to information averaged or estimated across all individuals in a study. In a two step approach the individual participant data are first analysed in each separate study independently and then synthesized using a suitable model for meta-analysis of aggregate data. With individual participant data an analytic standardisation across the studies and a direct derivation of the information requested is possible, independent of significance or how it was reported.

Since at that time the microbiological evaluation was the primary aim practically in all UTI studies, a single-blind design was considered sufficient, because the eradication (primary aim) could be determined in an objective manner. Nevertheless, from todays point of view the single-blind design could be considered as a weakness of the studies, which may have affected mainly the clinical outcome and some subjective adverse events. However, the non-inferiority of nitroxoline for the eradication of bacteriuria as compared to the controls, cotrimoxazole and norfloxacin, is fairly well established by this IPD meta-analysis, whereas the individual studies contributed each between about 20 and 30% to the final results. Another shortcoming of the study may be, that after the test of cure the patients were not followed for a longer time. Therefore no information is available concerning the relapse rate after the test of cure visit.

Since the final microbiological evaluation in UTI studies is usually not performed at the end of treatment, but rather after the antibiotic substances are completely eliminated from the urinary tract and uropathogens, still present, have had time to multiply to significant numbers, the usual ITT and PP analyses may benefit good results, when patients drop out with favourable results after the end of treatment visit. These patients are considered a microbiological cure in the ITT set (last observation brought forward) and are drop outs for the classical PP analysis. Thus, possible treatment failures will be not recorded, because usually the reasons for dropping out after the treatment visit remains unknown. Therefore, a modified PP set was evaluated, in which all patients dropping out before test of cure were considered treatment failure (worst case scenario). Only if the same results can be achieved in all three evaluation sets, the overall result (non-inferiority) can be taken as proven, which was established by this IPD meta-analysis for nitroxoline as compared to the controls.

The current guidelines do usually not recommend a longer treatment duration of an acute episode in women with recurrent UTI than in those with sporadic UTI any more. For cotrimoxazole and fluoroquinolones even a 3-day therapy is considered sufficient in acute uncomplicated cystitis, but for the usage of macrocrystal nitrofurantoin also a 5-day therapy is recommended in the current guidelines [11]–[13]. Therefore a 5-day therapy of nitroxoline 250 mg tid should also be recommended as one of the first line options for the treatment of acute uncomplicated UTI (cystitis). Since about half of the female patients were pre- and postmenopausal in the studies meta-analysed, the dosage regimen and duration for both female groups can be considered the same, because in current guidelines an acute cystitis in otherwise healthy postmenopausal women is also considered as uncomplicated [12].

## Conclusions

Nitroxoline used for more than 50 years for the treatment and prophylaxis of adult patients and children suffering from UTI has shown good clinical efficacy and safety as published in 26 uncontrolled, in two earlier controlled and in one large postmarketing observational study including a total of more than 11,000 patients. This first IPD meta-analysis of four so far unpublished, prospective, randomized, comparative clinical studies with a total of 466 female patients with sporadic or recurrent uncomplicated UTI has demonstrated non-inferiority (10% non-inferiority margin) between treatment with nitroxoline versus controls (cotrimoxazole, norfloxacin). With a 5-day (sporadic UTI) and 10-day (recurrent UTI) treatment with nitroxoline 250 mg t.i.d. eradication of bacteriuria could be achieved in over 90% of the patients. The safety of nitroxoline was comparable with that of the controls. Adverse events, mainly gastrointestinal disorders and only a few allergic reactions, were reported only in 9.4% patients treated with nitroxoline and 7.8% with the control drugs. Combining the vast clinical experience obtained from studies published earlier and non-inferiority of bacteriological efficacy with nitroxoline versus the controls as documented by this high quality IPD meta-analysis and considering the world wide increase of resistance of uropathogens against cotrimoxazole and fluoroquinolones, which has not been seen with nitroxoline despite clinical usage for more than 20 years, nitroxoline should be reconsidered as one of the first line antibiotics for the treatment of uncomplicated UTI.

## Authors’ information

1. Assoc. Professor of Urology of the Technical University Munich, Munich; priv. address: Karl-Bickleder-Str. 44c, 94315 Straubing, Germany, e-mail: kurt@nabers.de; 2. Statistical Consulting & Data Analysis, Schlehendornweg 24, 07751 Jena, Germany, e-mail: niggemann@p-wert.de; 3. Rosen Pharma, Kirkeler Str.41, 66440 Blieskastel-Niederwürzbach, Germany, e-mail: gisela.stein@rosen-pharma.de; 4. Em. Professor of Internal Medicine of the University of Jena; priv. address: Von-Hase-Weg 22, 07743 Jena, Germany, e-mail: guenter.stein@med.uni-jena.de

## Authors’ original submitted files for images

Below are the links to the authors’ original submitted files for images. Authors’ original file for figure 1 Authors’ original file for figure 2 Authors’ original file for figure 3

## Abbreviations

CFU: Colony forming units
CRF: Clinical report form
CTX: Cotrimoxazole
IPD: Individual patients’ data
MIC: Minimal inhibitory concentration
mMITT: Modified microbiological intention to treat
mPP: Modified per protocol
n: Number
NFX: Norfloxacin
NTX: Nitroxoline
P: Prophylaxis
PP: Per protocol
SMX: Sulphamethoxazole
T: Therapy
UTI: Urinary tract infection
cUTI: Complicated urinary tract infection
rUTI: Recurrent urinary tract infection
uUTI: Uncomplicated urinary tract infection
