# Supplementary figures and images for: Review of the literature and individual patients’ data meta-analysis on efficacy and tolerance of nitroxoline in the treatment of uncomplicated urinary tract infections
Source: BMC Infect Dis. 2014 Nov 27;14:628. doi: 10.1186/s12879-014-0628-7 (PMC4262220; doi:10.1186/s12879-014-0628-7)

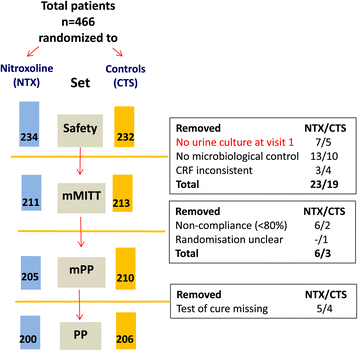

Supplement: Supplementary file 1 — Authors’ original file for figure 1 [file 12879_2014_628_MOESM1_ESM.gif]

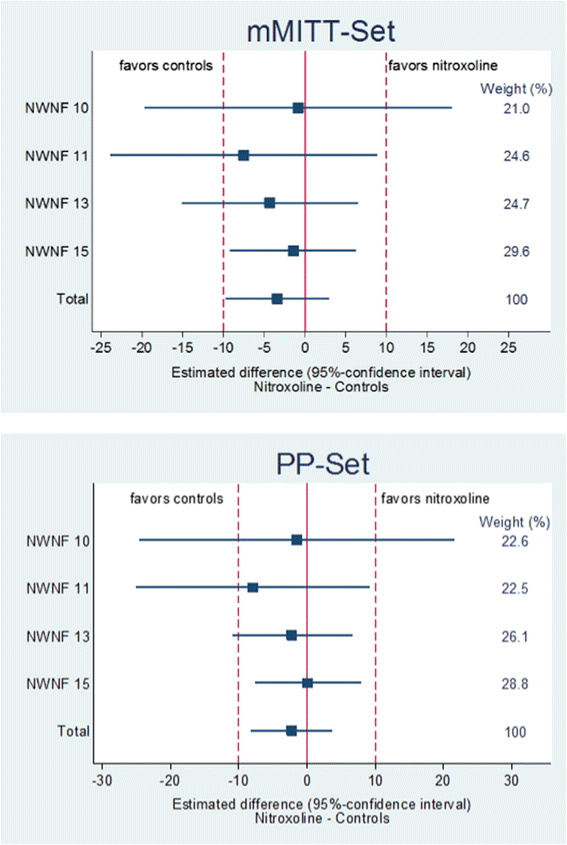

Supplement: Supplementary file 2 — Authors’ original file for figure 2 [file 12879_2014_628_MOESM2_ESM.gif]

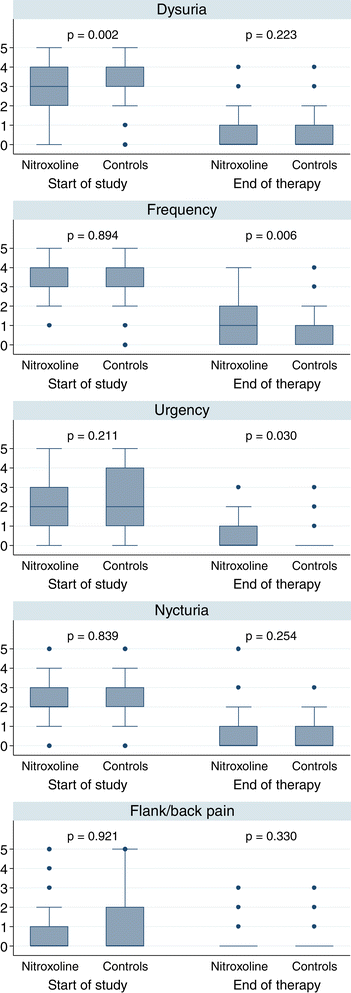

Supplement: Supplementary file 3 — Authors’ original file for figure 3 [file 12879_2014_628_MOESM3_ESM.gif]
